# Supplementary material for: SyNDI: synchronous network data integration framework
Source: BMC Bioinformatics. 2018 Nov 6;19:403. doi: 10.1186/s12859-018-2426-5 (PMC6219086; doi:10.1186/s12859-018-2426-5)
Supplement: Supplementary file 5 — Files of the example presented in Additional file 4. This additional file is a zip package that contains all files used in Additional file 4. Detailed descriptions of each of these files are presented in a README file included in this zip package. (GZ 30411 kb) [file 12859_2018_2426_MOESM5_ESM.gz › SyNDITools/Meme2Fimo/meme_4.9.0_4.tar.gz!/meme_4.9.0/website/html/help_filters.html]

MEME Suite - Sequence Filters 


|  |  |
| --- | --- |
|  | MEME Suite The MEME Suite web application requires the use of JavaScript   Javascript doesn't seem to be available on your browser.  - **shuffle sequence letters**    You can ask for the letters in each of your input sequences to be   shuffled. This can be useful for determining if the motifs found   using your (unshuffled) sequences are not statistically   significant. To determine this, compare the *E*-value (or score) for   the best motif/alignment from your original sequences with that   using the "Shuffle sequence letters" option. (Keep all   other parameter settings the same so that the comparison will be   valid.) Compare the best *E*-value (or score) for the first motif in   the original run to the best E-value (or score) for the first motif   in the shuffled run. If they are similar (or the *E*-value (score) in   the shuffled run tends to be better), then the motif is probably   not significant.     Repeat this process for each motif bearing in mind   that the earlier motifs are most likely to be significant.   Concluding that a motif is significant is more problematic. For   MEME, low *p*-value occurrences of the motif in many sequences and in   a conserved position relative to other motifs (or the ends of the   sequence) is evidence that it is statistically significant. You can   use the shuffle option to help determine what "low" is for the   particular set of sequences and parameters you are using. |
